# The Perceived Role of Occupational Therapists in Climate Change

**DOI:** 10.1177/00084174241259304

**Published:** 2024-06-11

**Authors:** Jiayi Du, Alexander Bird, Giovanna Boniface, Jeffrey Boniface, W. Ben Mortenson

**Keywords:** Occupational therapy, Climate change, Sustainability, Role, Occupational therapist, Planetary health, Changement climatique, durabilité, ergothérapeute, ergothérapie, rôle, santé planétaire

## Abstract

**Background.** In 2022, the World Health Organization (WHO) predicted that climate change would cause thousands of additional deaths per year from malnutrition, malaria, diarrhea, and heat stress alone between the years of 2030 and 2050. With such health consequences and environmental changes, climate change is impacting human occupations globally. However, there is a gap in the literature regarding the occupational therapists’ role in climate change, particularly in the Canadian context. **Purpose.** Our research aimed to explore what is the perceived role of occupational therapists in climate change and climate action from the perspective of Canadian occupational therapists and international experts. **Method.** This qualitative study used interpretive description methodology. We recruited 12 occupational therapists, including 4 research experts in the field. We conducted semi-structured interviews with each participant. Data were analyzed thematically. **Findings.** This study uncovered three themes that focused on the complex interconnections between climate challenges and climate actions that occupational therapists are wrestling with personally, clinically, and professionally. Specifically, this study emphasized the importance of supporting individual occupational therapists with their personal challenges, integrating climate actions into clinical practices, and incorporating climate change and climate justice into occupational therapy curricula and professional advocacy. **Implications.** The environment, including the planet's ecosystem, is a fundamental component in many models of occupational therapy practice. This research provides a rich understanding in the themes of occupational therapists’ perceptions of climate change and climate actions, particularly within a Canadian context.

## Résumé

**Description.** En 2022, l’Organisation mondiale de la santé (OMS) a prédit qu’entre 2030 et 2050, les changements climatiques entraîneraient des milliers de décès supplémentaires dus à la malnutrition, au paludisme, à la diarrhée et au stress thermique. Avec de telles conséquences sur la santé en plus des changements environnementaux, le changement climatique affecte les occupations humaines à l’échelle mondiale. Cependant, il y a des lacunes dans la littérature concernant le rôle des ergothérapeutes dans les changements climatiques, particulièrement dans le contexte canadien. **But.** Notre recherche vise à explorer le rôle perçu des ergothérapeutes dans le changement climatique et l’action climatique du point de vue des ergothérapeutes canadiens et des experts internationaux. **Méthodologie.** Cette étude qualitative a recours à une méthodologie de description interprétative. Nous avons recruté 12 ergothérapeutes, dont quatre chercheurs experts dans le domaine. Nous avons mené des entretiens semi-structurés avec chaque participant. Les données ont été analysées thématiquement. **Résultats.** Cette étude a permis de mettre en relief trois thèmes axés sur les interconnexions complexes entre les défis climatiques et les actions climatiques auxquelles les ergothérapeutes sont confrontés sur les plans personnel, clinique et professionnel Plus précisément, cette étude souligne l’importance de soutenir les ergothérapeutes individuellement dans leurs défis personnels, d’intégrer les actions climatiques dans les pratiques cliniques et d’intégrer les changements climatiques et la justice climatique dans les programmes d’ergothérapie et la défense des droits et intérêts professionnels. **Implications.** L’environnement, y compris l’écosystème de la planète, est une composante fondamentale de nombreux modèles de pratique de l’ergothérapie. Cette recherche offre une riche compréhension des thèmes qui ressortent des perceptions des ergothérapeutes à l’égard des changements climatiques et de l’action climatique, particulièrement dans le contexte canadien.

## Introduction

There have been increasingly dire warnings about the potential for mass extinction events because of human-induced climate change (Intergovernmental Panel on Climate Change, 2022). Climate change refers to long-term shifts in temperatures and weather patterns, with human activities, particularly the burning of fossil fuels, being the main driver since the 1800s (United Nations, n.d.). In 2022, the World Health Organization (WHO) predicted that climate change would cause approximately 250,000 additional deaths per year from malnutrition, malaria, diarrhea, and heat stress alone between the years 2030 and 2050 (WHO, 2022). Reagon (2020) indicated that climate change is an overwhelming risk to the current and future health of populations globally. Furthermore, there will be progressively more climate refugees due to global warming (Reagon, 2020). According to global-scale research findings, it is imperative for stakeholders at all societal levels to undertake measures to address climate change (WHO, 2022).

There is a growing demand to prioritize intersectoral approaches to address severe existential health risks from climate change (Butler, 2018). In 2021, The United Nations developed a Healthy Climate Prescription Statement (WHO & Global Climate and Health Alliance, 2022) in partnership with 102 different countries and 600 organizations representing over 46 million healthcare professionals, including the World Federation of Occupational Therapists (WFOT, 2021). This statement addresses how healthcare services around the world need to respond to climate change, such as call on governments to build sustainable health systems. Notably, but unfortunately, Canada is warming at a rate almost double that of the rest of the world, with northern regions of the country experiencing rates nearly triple the global average (Bush & Lemmen, 2019). In 2023, Canada experienced record-breaking wildfires, with more than 6,000 fires recorded by Natural Resources Canada which doubled the 1989 record (Natural Resources Canada, 2023). Lowe and Garfin (2023) identified exposure to low air quality, crisis-related media exposure, and increasing climate anxiety as immediate health consequences post wildfires. It is important to recognize that the Canadian government has released the first National Adaptation Strategy to build strong and resilient communities in the facet of climate change (2023).

Since Pereira suggested the connection between mental health and climate change from an occupational science perspective in 2008 (Pereira, 2008), there has been rising literature in occupational science and occupational therapy exploring climate change and sustainability. An occupational science scoping review indicated that occupational engagement in sustainability is impacted by a variety of factors, including culture, policy, economics, demographics, and history (Lieb, 2022a). Multiple international studies have also suggested the importance of extending the concept of occupation beyond human occupations and in connection with the entire ecosystem (Algado, 2023; Aoyama, 2014; Persson & Erlandsson, 2014; Ung et al., 2020). Additionally, Smith et al. (2020) have conducted scoping reviews focused on the specific connection between occupational therapy (OT) and environmental sustainability. To connect OT practice with eco-sustainability, Drolet and Turcotte (2021) and Drolet and Lafond (2022) have highlighted the importance of intergenerational occupational justice and ethical reflections. Lafond and Drolet have explored the implementation of sustainable practice among Quebec occupational therapist across micro, meso, macro environments (2021) and categorized sustainable OT practice into clinical practices, organizational practices, and social practices (2023). Relatively little is known about the perspectives of Canadian occupational therapists about climate change in the English literature. One of the only Canadian surveys in the English literature included responses from 81 occupational therapists of which more than half (54%) of the participants reported having moderate to no skills to integrate climate mitigation clinically (Chan et al., 2020). Given the current gaps regarding occupational therapists’ roles in climate change in the Canadian English literature, this research aimed to explore what is the perceived role of occupational therapists in climate change and climate action from the perspective of Canadian occupational therapists and international experts.

## Methods

This study recognized the significance of clinical context and application while exploring the role of Canadian occupational therapists in climate change. This study employed interpretive description as the primary methodology, embracing and highlighting the clinical knowledge and experiences of the participants (Thorne, 2016) and it is commonly used in practice-oriented research to generate knowledge of complex phenomena. This interpretive description study used semi-structured interviews as the primary means of data collection (Thorne, 2016). The study received approval from the University Behavioural Research Ethics Board (H22-01664) and is reported in Appendix 1 according to the Standards for Reporting Qualitative Research guideline.

### Participants

To take part in the study, participants needed to be either research experts with special interests in climate change within the context of OT or occupational therapists registered to practice in Canada. Given that climate change is a global challenge, involving research experts in the OT-climate change field from both Canada and the international community, along with Canadian occupational therapists, could further deepen our understanding of this topic. According to Thorne (2016), having multiple datasets could both add complexity to the comprehension of the topic and confirm conclusions from multiple perspectives. All participants were required to be fluent in English.

The recruitment consisted of two parts, with convenience sampling as the major approach. To recruit Canadian occupational therapists as participants, national and provincial Canadian OT associations were asked to send information about the study to their members. For three out of six research experts, the first two authors directly recruited them through emails linked to their previous publications. Additionally, the third author recruited both Canadian occupational therapists and research experts (remaining three) through social media posts.

### Data Collection

Demographic data were collected from all participants, including age, gender, ethnicity, area of practice/research, and years of practice/research. The interview guide contained open-ended questions aimed at exploring participants’ experiences, feelings, and perceptions regarding climate change and climate action within the realm of OT. Minor modifications to the interview guide were made based on the flow of the first interview. In recognition of our limited knowledge and experiences of climate change from an Indigenous perspective, we amended the interview guide to include follow-up questions to learn from participants who had worked closely with Indigenous communities. Interviews were approximately one hour and were conducted via Zoom. Generally, researchers and participants joined Zoom meetings from quiet and private physical spaces, including home and office rooms.

### Data Analysis

Interview recordings were transcribed verbatim by using the Zoom auto-transcription feature, and then manually checked and corrected by the first two authors. Data collection and analysis occurred simultaneously, following the idea that researchers and participants co-create understandings in interpretive description (Thorne et al., 2004, p. 5). To align with the methodology of interpretive description, broad-based codes were used in the early coding stage (Thorne, 2016). As the coding stage moved forward in this study, constant comparative analysis was applied: we compared all data pieces to theorize all possible relations (Thorne, 2016). Both broad-based codes and constant comparative analysis were conducted independently. Subsequently, the first two authors discussed and compared their analyses, aiming to develop a comprehensive understanding of the codes while considering different perspectives. Two researchers created analytic notes throughout the analysis process, which helped analysis move beyond thematic analysis and transition into more creative and interpretive realms of determining options for depicting patterns or meanings (Thorne, 2016). As the final product, a coherent professional, persuasive, and clinically applicable narrative was created. Through this final product along with researchers—participants co-creating understanding, we believe we have achieved theoretical sufficiency, which implies having reached a sufficient or adequate depth of understanding of the topic (Dey, 1999).

We employed several trustworthiness strategies. First, convenience sampling and having participants from different geographic locations provided rich complementary perspectives. Furthermore, all data collection involved at least two researchers. The first two authors participated in all stages of interviews and data analysis. The last author participated in and provided feedback on the first interview. Additionally, the last three authors provided feedback and guidance for the development of themes. Debrief sessions were part of the reflexivity strategy and acted as a way of collaborating all perspectives. To further promote reflexivity, pre-interview assumptions and post-interview reflections were recorded and debriefed with the team.

In terms of positionality, the first author is a Chinese female, the third author is a Caucasian female, and the second, fourth, and last authors are all Caucasian males. The first two authors were student occupational therapists between the ages of 25 and 35, with strong personal passion for the ecological environment. The last three authors were all experienced occupational therapists and university faculty members. Identified biases included assuming all participants shared similar extents of climate challenges from their macro contexts, such as political contexts and resource allocation. As these biases were revealed, follow-up questions around macro environments were added in the interview guideline to reduce researcher assumptions.

## Results

Demographic information for the twelve participants is summarized in Table 1.

**Table 1.** Interviewee's Demographics

Characteristics	Categories	Mean ± SD or N (%)
Age (years)		34 ± 5
Gender	Female	10 (83%)
	Male	2 (17%)
Years of practice		10 ± 7
Type of participants	Key informants-researchers	6 (50%)
	Occupational therapists	6 (50%)
Participant location	Canada	8 (67%)
	Canada-Central Provinces	4
	Canada-West Coast	2
	Canada-Northern territories	2
	US	3 (25%)
	UK	1 (8.3%)

Abbreviations: SD = Standard deviation.

To preserve participants’ anonymity, pseudonyms are used for each participant. Reference to international key informants’ countries of practice is included to provide meaningful political and practice context to their views and suggestions.

Through our analysis, three themes have emerged, which were “Climate change is real”: Negotiating climate change personally, “There's no occupational being without the planet”: Struggling with climate change clinically, and “We need to use our voice”: Confronting climate change professionally.

### “Climate Change is Real”: Negotiating Climate Change Personally

This theme explored the individual views, feelings, and actions toward climate change, as well as the impacts of climate change on individual occupational therapists. All participants reported they did not only believe in climate change but also cared about reducing its impact. Chloe, an occupational therapist from the West Coast of Canada, described climate change as “a really big issue […] one that's been present in [her] life, for as long as [she] can remember.” Sophia, an occupational therapist from the eastern Canada with an environmental science research background, shared that “[climate change] ties a lot with being a global citizen, and not following ‘it's not in my backyard’ mentality.”

Multiple participants shared their personal encounters with climate change. Kai, an American OT researcher, stated that “I think [climate change] is important […] the state where I live is constantly experiencing drought […] wildfires […] and frequent earthquakes too.” Max, identified as both occupational therapist and Canadian researcher who had worked with rural communities in the northern region of Canada, experienced first-hand wildfires, and extreme heat. Max tied both his views and feelings on climate change with his experiences, “It's awful that I always find [climate change] in the back of my head on any given day […] during really severe heat wave seasons, [my climate anxiety] is worse.” In addition to direct feelings about climate change, participants described their feelings related to climate actions and responses. Emily, an American OT researcher, battled with her climate guilt:

It was hard at first [to deal with this guilt], because I want to have a low carbon footprint. But also, I want to make a living, so I shouldn’t have to feel guilty about driving [to see clients] and making a living by using my degree.

Similarly, Olivia, an OT researcher from the southern US expressed her fears of “missing the mark to take climate actions.” Many Canadian occupational therapists also resonated with the same feelings of climate guilt and fear of not doing enough. However, different from all other participants, Olivia was constantly under political pressure whenever she took a stance for climate change as “where [she] located geographically, [climate change] is not even always a topic of acceptance.”

Despite the complexity of these feelings, participants shared their coping strategies. Max said, “I try to cope with climate anxiety […] by going for walks and staying physically active.” Similarly, Victoria, an occupational therapist who had worked closely with Indigenous communities on the West Coast shared “if I want to express tough emotions, I will go to the body of water that I live next to.” In addition to emotional coping strategies, participants reported other climate actions they are taking. Evelyn, an occupational therapist from a West Coast island, shared that “I moved […] so that I wouldn’t have to commute [by driving]” to reduce the carbon footprint. Additionally, Evelyn liked to “recycle clothes and mend them” and she shared this “hobby” with her colleagues. Similarly, Sophia shared “one thing my colleagues have really picked up on is I use my own cutlery.” Besides lifestyle-climate-actions, Kai described his climate action as “[creating] public outreach contents on climate change and sustainability.” Witnessing the impacts of his work, Kai wished to “focus more on advocacy” and “incorporate [climate change] into more events.” Not many Canadian participants focused on public outreach events, but they also identified future personalized goals around climate change. Chloe stated, “I will continue to work on […] types of things that I can do in my day-to-day to lower my climate impact.” Victoria wished to “listen more to the lived experiences [of vulnerable communities facing climate challenges]” and “become [more critical while looking at] research [because] research hasn’t included a lot of diverse populations.” Amelia, an occupational therapist working with rural and Indigenous communities in northern Canada, wished to continue to grow her community connections, as she shared “these Indigenous communities that are so full of knowledge, art, creativity, language, and adventure are ones that I hope I can continue to serve in the ways that they want me to.”

### “There's no Occupational Being Without the Planet”: Struggling with Climate Change Clinically

The second theme illustrated the effects of climate change on OT practice and ways that current and future practice could respond to climate change. In recognition of how people and their environments are inextricably linked, Charlotte, an occupational therapist from eastern Canada, indicated “There's no occupational being without the planet. Every single area of practice will have to somehow find their contribution.” Amelia described climate change as a contextual layer to her practice, “it's another layer that I look through […] when seeing people.” Grace suggested “a clean environment is part of [Indigenous] health, it's part of their wellbeing.” Additionally, participants tied climate change with occupations. Emily (US) suggested “all daily occupation has an environmental footprint.” In contrast, our Canadian participants focused on occupational disruptions as a result of climate change. Max said, “when it comes to climate change […] people's occupation is disrupted in some way.” Amelia provided an example of the loss of traditional hunting among northern Indigenous communities: “people [navigate] through the ice all the time [to find food resources] and now get lost because of [the melting ice] from climate change.”

Along with disrupted occupations as a result of changing climatic conditions, participants shared other climate challenges that their clients were confronted with. Chloe, with her practice focusing on youth, witnessed rising “eco-anxiety” among youth. Olivia suggested that “we’re going to see a lot of more chronic illness as a result [of climate change].” Furthermore, many participants reported witnessing displacement during their clinical practice. Amelia provided an example:

Some of the Indigenous communities [..] are very remote, and there's been a lot of forest fires. Because they’re so rural, remote, and small, there’re not as many fire systems to keep those forest fires in-check. So, a lot of these small communities either burn to the ground or people get evacuated.

In addition to climate challenges that participants’ clients were facing, participants reported barriers that they are facing first-hand. Max voiced that “[climate actions at his workplace] seems […] insufficient, and sometimes I do wonder if it's just a lip service.” Grace and Sophia both shared that the green initiatives at their workplaces are too little to notice or access. Additionally, Emily stated she couldn’t use telehealth to minimize driving because “in the US, we don’t get reimbursed for that.” (Noted that none of the Canadian participants mentioned insurance as a barrier to utilize telehealth.) Meanwhile, participants working with rural communities struggled with overall funding and support. Amelia summarized this experience as “usually the governments in the south [where major cities located] that fund all of these projects and they do not take into account people's personal experiences because it's difficult to quantify in their eyes.”

Despite struggling with the effects of climate change clinically and constantly experiencing barriers, participants wished to incorporate climate actions into OT practice. Charlotte suggested OT practice to view climate effects on “societal economic health and societal mental health” in addition to physical health. Isabella (UK) provided similar recommendations for OT practice as the Canadian participants:

Being physical and mental health orientated is important in terms of seeing the whole person and thinking how it affects people's climate anxiety, and as well as the physical problems of people who have been displaced or had homes destroyed.

Emily (US) suggested “[using] ethical reflection […] to figuring out what to do in [each] situation because OT is so complex and so client centered.” Similarly, Canadian therapists such as Max and Charlotte also wished occupational therapists to be “more reflective” in clinical practice.

More specific recommendations on how to incorporate climate change into OT clinical practice are summarized below in Table 2.

**Table 2.** Recommendations for Clinical Climate Actions in OT Practice

Categories	Focus	Specific recommendations
Areas of practice that occupational therapist has a direct role in climate change	Climate anxiety in youth	“The best practice [for climate anxiety among youth] is to look at […] what [people] are passionate about in their circle of influence and like their current environment.” Chole (Canada)
	Disaster management	“Disaster management is a big thing.” Emily (US)
	Equipment recommendations	“Making sure [everything is] from a proper source, that we’re sourcing them from ethical companies where that we know the products come from.” Isabella (UK) “Encourage the patients once they don’t use [durable equipment] anymore to donate or recycle.” Kai (US)
Approaches that embedded within the role of occupational therapist across settings	Interdisciplinary collaboration	“Connecting interdisciplinary training […] with people who are involved in like the carbon offset and building design when we’re looking at home modifications and technologies.” Evelyn
	Innovation	“Utilizing social media reach.” Kai (US) “We need to keep brainstorming.” Charlotte (Canada)
	Active listening and validation	“Listen to people when they are experiencing barriers to their activities of daily living due to climate change, to really acknowledge that the problem is real and to support them and finding interventions to overcome it.” Amelia (Canada)
	Exploring environmental stewardship	“Sometimes I work with clients on learning how to sort and recycle soft plastics, bottles, cans, and glasses […] One of my clients never walks in nature and that was his first goal […] now he wants to do environmental stewardship in the local park.” Evelyn (Canada)

### “We Need to Use Our Voice”: Confronting Climate Change Professionally

The last theme explored climate and systemic challenges that occupational therapists confronted professionally, as well as recommendations for future systemic and professional actions. Participants recognized systemic barriers in relation to climate change. Victoria suggested, “capitalism, white supremacy, and colonization [are prominent in the system], and all these kinds of [factors] have not treated the earth in reciprocal relationship.” American participants also shared similar views towards the current systemic oppression. Olivia said, “we are going to see a lot of disparities [from] systems of oppression […] there's such a disproportion of who's paying the price for [climate change].” Emily felt under the current systemic structures, “corporations don’t necessarily have to be responsible for the [huge amount of] carbon dioxide [they emit].”

Systemic barriers had challenged some participants to face devastating effects of climate change. Max shared, “The heat wave was really devastating […] for folks who are unhoused and who struggle with their mental health. There were a number of clients who died, and I think the system failed them.” Although Olivia is located in the US, she also emphasized housing inequity in her professional experiences with floods and tornadoes. She felt, “[Our profession is] behind with anything that centers justice, and I think climate change goes alongside.” Amid confronting these tragic events, many participants voiced their uncertainty. Chloe shared that, “Being on a professional level, I haven’t exactly figured out what to do.” Similarly, Amelia felt “[I] just don’t have the capacity to make big developmental choices [related to climate] right now.”

Globally, participants commonly shared there were limited climate guidelines supporting their clinical practice. Charlotte stated, “At least [the WFOT] identify [climate change] as an issue, and [Canadian associations] need to do something about it.” Moreover, Isabella said that guidelines on sustainability from British OT associations were “very broad ones” and wished to be more specific. Kai shared that “American [OT association] is just catching on what [UK] is doing.” Being aware of the actions of British and American OT associations and with the lack of guidelines in Canada, Max urgently called OT associations and regulatory organizations to “prioritize developing practice standards to be responsive to the effects of climate change. Do not keep our heads in the sand in the ways that perhaps we have been doing in the past.” Additionally, Amelia wished OT regulatory organizations to “acknowledge and add to the cultural safety part regarding how Indigenous communities are being impacted by climate change is crucial.” Likewise, Emily (US) suggested OT regulatory bodies focus on “the intragenerational and intergenerational occupational justice framework,” which was developed by a Canadian OT in collaboration with another Canadian OT and two OTs from France, when they develop climate guidelines.

In addition to professional guidelines, participants commonly highlighted the need to include climate change in the OT curriculum. Sophia suggested, “If [climate change] doesn’t enter the curriculum, I don’t think a lot of our new practitioners will have that mindset.” Specific suggestions on curriculum changes are summarized in Table 3, with four focus areas.

**Table 3.** Enhancing Climate Education in OT

Focus	Recommendations
Climate literacy—amount of climate information to include	“The more we bombard [OT students] with information, the more likely they’ll be to get on board with everything.” Emily (US)
The importance of curriculum change	“I think the textbook will be a short-term change. I think the longer-term change will be incorporating climate change in our curriculums.” Kai (US)
Context to include	“The [OT curriculum] should include more of social determinants of health […] and occupational deprivation.” Grace (Canada) “[the curriculum should] talk about entrepreneurship because this is a huge part of building confidence, and […] independence.” Charlotte (Canada) “[Including] more about colonization and reconciliation in OT.” Victoria (Canada)
More OT research	“We need to know how to prevent [effects of climate change] in health care versus being reactive.” Grace (Canada) “More opportunities to get funding or to get supports for different [climate] initiatives or research initiatives.” Sophia (Canada)

Besides professional guidelines and curriculum changes, many participants wished to see more advocacy from the profession. Charlotte shared, “if the next 100 years of OT is going to strive, […] we need to use our voice, we need to be confident.” Additionally, Charlotte highlighted the importance of our profession to advocate for climate actions in collaboration with other professions, “we need to take on that prevention role collectively […] and not too many health providers are in that prevention zone.” To provide further direction for collaboration in collective advocacy, Evelyn suggested, “in Canada, it would be key to collaborate with Indigenous peoples because they know this land the best.” Likewise, many participants suggested the power of building connections and communities. Sophia shared that “[climate advocacy happens through] being connected with people and having these conversations.” In addition, Emily (US) suggested that “in order to become a little bit more sustainable, we need people to […] be willing to help each other in their communities more.” Emphasizing the need for collective action to make a positive climate impact, Chloe highlighted that “it's not on the individual to save the world.”

Participants across three countries all emphasized the need for collective professional advocacy. Olivia (US) stated, “I would love to see OT as a profession looking to solve some of those problems.” Isabella (UK) suggested the limitation of bottom-up approaches during climate emergencies. Victoria gave a similar example:

If that came from a top-down thing, […], the hospital is required to do this from a legal perspective, it would happen. But when there's one person saying, hey, don’t forget to recycle, it just gets shut down so quickly.

Lastly, Charlotte, Max, and Olivia (US) all suggested the urgency for our profession to take climate action. Charlotte raised a question to the profession, “Changing systems isn’t easy because it often happens slowly. But we live in a time where we don’t have the luxury of waiting 40 years for change, right?”

## Discussion

The purpose of the study was to explore the perceived roles of occupational therapists in climate change and climate action from both Canadian occupational therapists and international experts. Overall, the perspectives from our international experts aligned closely with those of the Canadian participants, re-emphasizing that climate change is an urgent global issue. The results of this study uncovered the complex interconnections between climate effects and climate actions that occupational therapists are experiencing across the personal level, clinical level, and professional level. On a personal level, considering participants’ climate anxiety, first-hand experiences with extreme climate events such as wildfires, and structural barriers such as lack of guidance and support that they are facing, the results emphasized the importance of deindividualizing climate burdens, including shame and guilt of fearing not doing enough for the climate as an individual. This resonates with Hyman's reflection and call for reflections on the responsibility of climate, which suggests the importance of recognizing limited efficacy in individual choices and focusing on larger scale solutions in addition to individual sustainable efforts (Hyman, 2020). Furthermore, this study uncovered the need to develop resources, organizational changes, and systemic changes to support individual occupational therapists, which resonated and aligned with previous findings (Kreslake et al., 2018; Lafond & Drolet, 2021).

On a clinical level, the need to support physical and mental health and well-being in the face of climate change aligned with the Canadian National Strategy plan (Government of Canada, 2023). Specifically, participants emphasized the crucial role of occupational therapists in supporting rising climate anxieties among clients, especially youth. Their challenges align with findings from both Lowe and Garfin (2023) and Hickman et al. (2021). Hickman et al. (2021) revealed that climate anxiety affects young people daily functioning worldwide, including reported feelings of hopelessness and perceived future as frightening. The increasing prevalence of climate anxiety among young people calls for effective support mechanisms, highlighting the crucial role of occupational therapists, as suggested by Parsonage-Harrison et al. (2023). Falardeau (2021) and Hurtubise (2022) have explored the specific roles of occupational therapists in climate anxiety management, including engaging client participation in ecofriendly meaningful occupations and providing emotional validation. Additionally, the findings support the involvement of occupational therapists in disaster management which aligned with the occupational therapist's role in disaster risk reduction and management promoted by WFOT (2022). Furthermore, the use of lifecycle assessment (Brussau et al., 2019), a tool to identify sustainable source, materials, and recycling methods, to guide equipment recommendations promotes sustainability in clinical and organizational practices, echoing ideas presented by Lafond and Drolet (2023) regarding more sustainable management of equipment, supplies, and materials in OT practice. It is important to note that the Association Nationale Française des Ergothérapeutes (ANFE), the Réseau pour le Développement Durable en Ergothérapie (R2DE) and the Communauté Ergothérapique Engagée pour L’équité et L’environnement (C4E) have developed a set of practical tools to support OT clinical and organizational practices to respond to the urgency of the climate crisis (C4E, 2022; R2DE et al., 2017).

On a professional level, participants identified systemic barriers associated with capitalism and colonialism. As a result, they recommended that the profession prioritize justice and include climate justice in OT frameworks, curriculum, and clinical interventions. Furthermore, this recommendation was focused on fostering a forward-thinking mindset for the betterment of future generations and the future trajectory of OT practice. This aligns with the concept of intergenerational occupational justice, proposed by Drolet et al. (2020), which emphasizes using a justice lens to view climate change in the context of OT and highlights the connection between climate change and occupational rights, emphasizing the importance of protecting occupational rights for future generations (Kiepek, 2023; Lieb, 2022b). Participants emphasized the importance of including and prioritizing Indigenous perspectives in OT models and curriculums, aligning with the global climate initiatives with Indigenous communities led by United Nations Educational, Scientific and Cultural Organization (UNESCO). Specifically, UNESCO addresses the intersection of Indigenous and local knowledge with climate change through its climate policy briefs (UNESCO, 2018), and it has launched the Local and Indigenous Knowledge Systems Program to promote the recognition and utilization of Indigenous knowledge in climate policy (UNESCO, 2023). Kiepek (2023) has also suggested that occupational justice includes honouring the interdependence of species and it is part of the OT professional responsibility to honor Indigenous worldviews and welcoming the potential for Western conceptualisations of occupation to be transformed. This perspective acknowledges the deep connection between Indigenous communities and the environment, recognizing their traditional knowledge and practices as valuable resources for addressing climate change and promoting sustainability.

The findings from this study emphasized the importance of curriculum changes to equip future occupational therapists to take further climate actions in their practice. Wagman et al. (2020) also emphasized on the significance of explicitly including sustainability in the OT curriculum and suggested continuous reflection about course content and research in relation to sustainability and sustainable development. However, Benevides et al. (2015) proposed that curriculum revision can improve perceived knowledge and skill, but suggested other factors, such as the system structure, would influence the clinical application of this knowledge post-graduation. This highlights the importance of advocacy, one of our other findings, from the OT bodies to make systemic changes. Additionally, most of our participants shared similar professional values and personal values regarding climate change. This contrasts with a previous OT climate study in Canada, which identified a discrepancy between personal and professional values in climate change (Chan et al., 2020). Potential reasons for this discrepancy within Chan et al.'s (2020) study could be attributed to an increase in natural disasters since 2020 when the earlier research was published, along with the rise of climate initiatives within the Canadian OT community, such as podcasts, professional practice publications, and conference discussion sessions on climate change.

Overall, these dynamic and complex interconnections align with the complexity of people, their environments, and the interactions in between that are highly emphasized in the Kawa River Model (Iwama, 2006; Teoh & Iwama, 2015). In addition to centering the experience of the person (usually the client) in the Kawa River Model, our findings suggest there are dynamic therapist-client interactions related to climate challenges. Specifically, findings suggest that climate change impacts OT practice and magnifies climate challenges and the effects of systemic barriers. On one hand, these barriers challenge occupational therapists from taking further climate actions or responding to consequences from climate change in their practice. On the other hand, resources and skills facilitate occupational therapists to overcome climate challenges and take further climate actions. Additionally, because of climate actions, climate change can be potentially mitigated, and its consequences can be altered through OT practice. This finding highly aligned with the concept of interactionism where human perceptions and choices are based on their interactions with the social and physical environment (Blumer, 1969). This dynamic interconnection of climate experiences among occupational therapists aligned with the interdependence between human occupations, lifestyle diseases, and climate change is validated by previous studies (Garcia Diaz & Richardson, 2023). This inextricable connection between occupation and the environment can also be explained by transactionalism, where the ongoing process of interaction between a person and their physical and social environment is highly emphasized (American Psychological Association, 2023). This resonates further with the perspective on occupations suggested by Dickie et al. (2006), where occupation is a mode through which human beings, as organisms-in-an-environment-as-a-whole, function as a complex totality, rather than a type of self-action arising solely from within individuals.

This research had several limitations. Although we believe we have reached theoretical sufficiency in this exploratory study, further interviews could potentially identify additional insights. Participants who engaged with the study were already passionate about the topic of climate change, potentially leading to biased responses and views. It is also important to note that four of the twelve participants were not Canadian, and thus the Canadian sample remains small. Additionally, this study only included English speaking occupational therapists and research experts. Instead of semi-structured interviews, future studies could use a survey or mixed-methods approach to achieve a broader participant representation.

With rising climate effects and calls for climate actions, there is an urgent demand for OT research in this area. At the personal level, further research could investigate the specific support that individual therapists would need to take climate actions and face climate challenges. In recognition that climate change is a collective effort, studies could also explore the perspectives of climate change deniers and develop transformation strategies. Due to the urgency of climate crises, it is important to encourage all therapists who see the problem to take actions in the meantime. At the clinical level, future studies could investigate effective methods for occupational therapists to incorporate climate change into their clinical assessments, interventions, and overall practice. On a professional level, further research could support the exploration and development of climate-related OT models for OT curricula and clinical related climate action guidelines. Furthermore, both OT and occupational science research could further support calls for climate actions on both systemic and political levels, collaborate with other professions, and allyship with Indigenous communities.

## Conclusion

This study explored the role of Canadian occupational therapists in climate change and action across personal, clinical, and professional levels. At a personal level, the study revealed the need to support individual occupational therapists with their personal challenges, especially by deindividualizing climate burdens. On a clinical level, the study identified the crucial need for occupational therapists to take climate actions through assessments, interventions, equipment recommendations, and other organizational sustainable approaches. At a professional level, this study emphasized the need to include climate change and climate justice in OT curricula, models, and professional advocacy. Additionally, the study highlighted the complex and inextricable connection between occupation, individual (both occupational therapists and their clients), and the environment (including ecological environments and environmental barriers). Thus, this study aims to call for further climate actions within OT practice and higher-level changes for such actions to take place.

## Key Messages

- Canadian occupational therapists are experiencing dynamic climate effects across the personal, clinical, and professional level.
- There is an urgent need for occupational therapists to engage in climate action on clinical levels. This includes supporting clients with climate anxieties, disaster management, and promoting sustainable equipment use, as well as environmentally friendly organizational approaches.
- There is an urgent need for occupational therapists to engage in climate action on professional levels, including explicitly addressing climate change with occupational therapy curriculum, research, and political advocacy.
